# Inhibition of mammalian target of rapamycin improves neurobehavioral deficit and modulates immune response after intracerebral hemorrhage in rat

**DOI:** 10.1186/1742-2094-11-44

**Published:** 2014-03-06

**Authors:** Qin Lu, Lu Gao, Lijie Huang, Linhui Ruan, Jianjing Yang, Weilong Huang, Zhenxing Li, Yongliang Zhang, Kunlin Jin, Qichuan Zhuge

**Affiliations:** 1 Department of Neurosurgery, First Affiliated Hospital of Wenzhou Medical University, Wenzhou, Zhejiang, China; 2 Zhejiang Provincial Key Laboratory of Aging and Neurological Disease, Wenzhou Medical University, Wenzhou, Zhejiang, China; 3 Department of Pharmacology and Neuroscience, Institute for Aging and Alzheimer’s Disease Research, University of North Texas Health Science Center at Fort Worth, Fort Worth, TX, USA

**Keywords:** ICH, mTOR, Rapamycin, Outcome, Immune response

## Abstract

**Background:**

Mammalian target of rapamycin (mTOR), a serine/threonine kinase, regulates many processes, including cell growth and the immune response. mTOR is also dysregulated in several neurological diseases, such as traumatic brain injury (TBI), stroke, and neurodegenerative disease. However, the role of mTOR in intracerebral hemorrhage (ICH) remains unexplored. The aims of our study were to determine whether inhibiting mTOR signaling could affect the outcome after ICH and to investigate the possible underlying mechanism.

**Methods:**

A rat ICH model was induced by intracerebral injection of collagenase IV into the striatum, and mTOR activation was inhibited by administration of rapamycin. mTOR signaling activation was determined by western blotting. Neurobehavioral deficit after ICH was determined by a set of modified Neurological Severity Scores (mNSS). The levels of CD4+CD25+Foxp3+ regulatory T cells (Tregs) and cytokines were examined using flow cytometry and ELISA, respectively.

**Results:**

Our results demonstrated thatmTOR signaling was activated 30 minutes and returned to its basal level 1 day after ICH. Increased p-mTOR, which mean that mTOR signaling was activated, was predominantly located around the hematoma. Rapamycin treatment significantly improved the neurobehavioral deficit after ICH, increased the number of Tregs, increased levels of interleukin-10 and transforming growth factor-β and reduced interferon-γ both in peripheral blood and brain.

**Conclusions:**

Our study suggests that mTOR improves ICH outcome and modulates immune response after ICH.

## Introduction

Stroke is the second leading cause of death worldwide [1], and is one of the major causes of morbidity and mortality. Intracerebral hemorrhage (ICH), a lethal type of stroke, accounts for 20% of all strokes [2], and occurs in about 50–60% of East Asians [3]. Currently, no specific therapies are available that improve the outcome after ICH. Surgical treatment of ICH, such as surgical removal of the hematoma, is primarily supportive, but the clinical outcome is poor, increasing the potential extensive burden for the family and society. Thus, the problem of improving neurological disability after ICH is still needs to be resolved.

In the early 1990s, the two yeast genes *TOR1* and *TOR2* were discovered in the budding yeast *Saccharomyces cerevisiae* by a group performing genetic screens for the toxic effects of rapamycin [4]. Mammalian target of rapamycin (mTOR) was then quickly identified, purified, and cloned [5,6]. mTOR is an evolutionarily conserved serine/threonine kinase that plays a key role in the regulation of cell growth and metabolism [7]. The mTOR pathway integrates diverse environmental signals to regulate many processes including autophagy, ribosome biogenesis, transcription, translation, and cytoskeletal organization [8,9]. In addition to cancer, dysregulation of mTOR activity has been found in neural diseases, such as epilepsy [10], Parkinson’s disease [11,12], Alzheimer’s disease [13], and traumatic brain injury (TBI) [14]. Chen *et al*. found in a model of TBI that mTOR was significantly phosphorylated from 30 minutes to 24 hours after TBI. Considering the role of mTOR in regulating mRNA translation for cell growth, dendritic arborization, and hippocampal synaptic plasticity, its activation may contribute to the deficits in learning and memory after TBI [14]. However, the questions of whether mTOR is also activated after ICH, and whether activation of mTOR related to neurobehavioral deficits still need to be determined.

An increasing number of studies have shown that inflammatory responses are deeply involved in the progression of ICH-induced brain injury [15,16], which may be mediated by: 1) cellular components including local microglia, lymphocytes, and leukocytes, and 2) molecular components including pro-inflammatory and anti-inflammatory cytokines. These two major components interact with each other and contribute to the outcome of stroke [17,18]. Thymus-derived CD4+CD25+Foxp3+ regulatory T cells (Tregs) are known to be neuroprotective in stroke [19] by modulating the function of effector T cells and secreting anti-inflammatory molecules such as interleukin (IL)-10 and transforming growth factor (TGF)-β [20,21]. Recent studies have shown that mTOR is proving to be a vital link between immune function and metabolism, and inhibition of mTOR by rapamycin promotes the production of CD4+CD25+Foxp3+ Tregs [22,23]. Thus, the question arises as to whether inhibition of mTOR might also improve neurological disability by modulating the immune response after ICH.

In order to address the issues described above, we first investigated alterations of the mTOR signaling pathway around the hematoma after ICH, and then examined the effect of rapamycin treatment on neurological deficits after ICH. Finally, we explored the effect of rapamycin administration on immune response in a rat model of ICH.

## Materials and methods

All experimental procedures were approved by the Animal Care Committee of the Wenzhou Medical University.

### Animals

In total, 102 male Sprague–Dawley rats, weighing 280 to 320 g, were randomly divided into eight groups. Group 1 (n = 9) was the normal control group and did not receive any treatment; group 2 was the ICH group (n = 26), which received collagenase injection at 30 minutes, 1 hour, 4 hour, 1 day, and 3 days after induction of stroke; group 3, the AU group (n = 8), received injection of autologous blood; group 4, the PBS group (n = 6), received PBS (containing 2.5% DMSO, 5% polyethylene glycol 400 and 5% Tween 80) 1 hour after ICH; groups 5 to 8 were treated with rapamycin (n = 53) at concentration of 50 (n = 11), 150 (n = 23), 250 (n = 11), and 500 ug/kg (n = 11), respectively, via intraperitoneal injection.

### ICH model

The experimental ICH model was induced by intrastriatal collagenase injection, as previously described by Rosenberg *et al*. [24]. Briefly, rats were anesthetized using 4% chloral hydrate and then placed in a stereotaxic frame (KOPF, California, USA) After the bregma was exposed, a borehole (1 mm) was drilled 3 mm lateral to the bregma and then a 30-gauge needle was inserted through the bore hole into the striatum (5 mm ventral from the skull surface).

For the collagenase-injection model, 1 μl collagenase type IV (0.25 IU/μl) was injected into the striatum over a period of 5 minutes to induce ICH. The needle was allowed to remain *in situ* for another 5 minutes before it was removed.

For the autologous blood-injection model, 50 μl autologous blood, which was withdrawn from the caudal artery, was injected into the striatum in a double-injection manner, as described previously by Deinsberger *et al*. [25]. During injection, the temperature was monitored and maintained at 37 ± 0.5°C. Animals recovered at room temperature with free access to food and water under a 12-hour light/dark cycle.

### Rapamycin

Rapamycin (Merck Millipore, Darmstadt, Germany) was dissolved in DMSO (5 mg/ml) and stored at -20°C. Before injection, rapamycin was diluted with PBS containing 5% polyethylene glycol 400 and 5% Tween 80. The ICH rats received a single intraperitoneal injection of rapamycin 1 hour after collagenase administration, and the doses of rapamycin used were 50, 150, 250, and 500 μg/kg for groups 5 to 8, respectively.

### Western blot analysis

In order to assess the profile of mTOR activation after ICH, rats were euthanized at 30 minutes, 1 hour, 4 hours, 1 day, 3 days, or 7 days after ICH. The brains were removed, and the left (ipsilateral) and right (contralateral) hemispheres were separated. The tissues were then homogenized in RIPA buffer (Thermo, Rockford, USA) containing 10 μl of protease inhibitors and phosphatase inhibitors (Thermo) per 1 ml RIPA. The protein concentration was determined by bicinchoninic acid assay (Thermo). Equivalent quantities of protein (50 μg per lane) were separated in 6 to 15% SDS-polyacrylamide gels, and transferred to PVDF membranes. After blocking, the membranes were incubated overnight at 4°C with the following primary antibodies: rabbit anti-mTOR monoclonal antibody, rabbit anti-p-mTOR (Ser 2448) polyclonal antibody, rabbit anti-phospho -p70S6 (Thr 389) monoclonal antibody, rabbit anti-p70S6 monoclonal antibody, rabbit anti-tubulin polyclonal antibody (1:1000) (all from Cell Signaling Technology). After washing three times in TBS buffer with 0.1% Tween (TBS-T), membranes were further incubated with HRP-conjugated anti-rabbit IgG secondary antibody for 1 h at room temperature, and the bands were visualized by enhanced chemiluminescence (Thermo, USA) and X-ray film(Thermo, USA) exposure. Quantity One software (BioRad) was used to detect the band intensities. To ensure reproducibility, all experiments were repeated at least three times.

### Behavioral testing

For behavioral testing, rats were assessed before ICH and at 1, 3, 7, and 14 days after ICH by an investigator who was blinded to the experimental groups using a set of modified Neurological Severity Scores (mNSS) [26,27]. The mNSS is a complex behavioral test including motor, sensory, reflex, and balance tests. In this test, the scores ranged from 0 to 18, where 0 was considered normal and 18 was maximum deficit.

### Flow cytometry

At 3 days after ICH, control rats (n = 6), ICH rats (n = 8), and rats treated with 150 μg/kg rapamycin (n = 10) were euthanized and perfused with saline. The blood and brain of each rat were collected, and the monocytes were separated as described previously [28]. Cells were stained with antibodies (BD Biosciences): FITC–anti-rat-CD4, APC-anti-rat-CD25, and PE-anti-rat-FoxP3. Stained cells were analyzed by blinded evaluators using a flow cytometer (FACSCalibur; BD Biosciences, USA) and Flowjo 7.6 software.

### ELISA

To test the inflammatory response after rapamycin treatment, colorimetric ELISA kits were used to detect the following cytokines in serum and brain protein extract: rat IL-10, rat interferon (IFN)-γ, and rat TGF-β (all R&D Systems, USA). For each ELISA analysis, 40 μl of sample was used without dilution in accordance with the manufacturer's instructions.

### Statistical analysis

Data areshown as means ± standard deviation (SD). Statistical differences between normal control group and other groups were compared using student T-tests. Statistical significance was defined as *P<0.05 and **P<0.001.

## Results

### Alteration of mTOR signaling pathway after ICH

**Figure 1:**
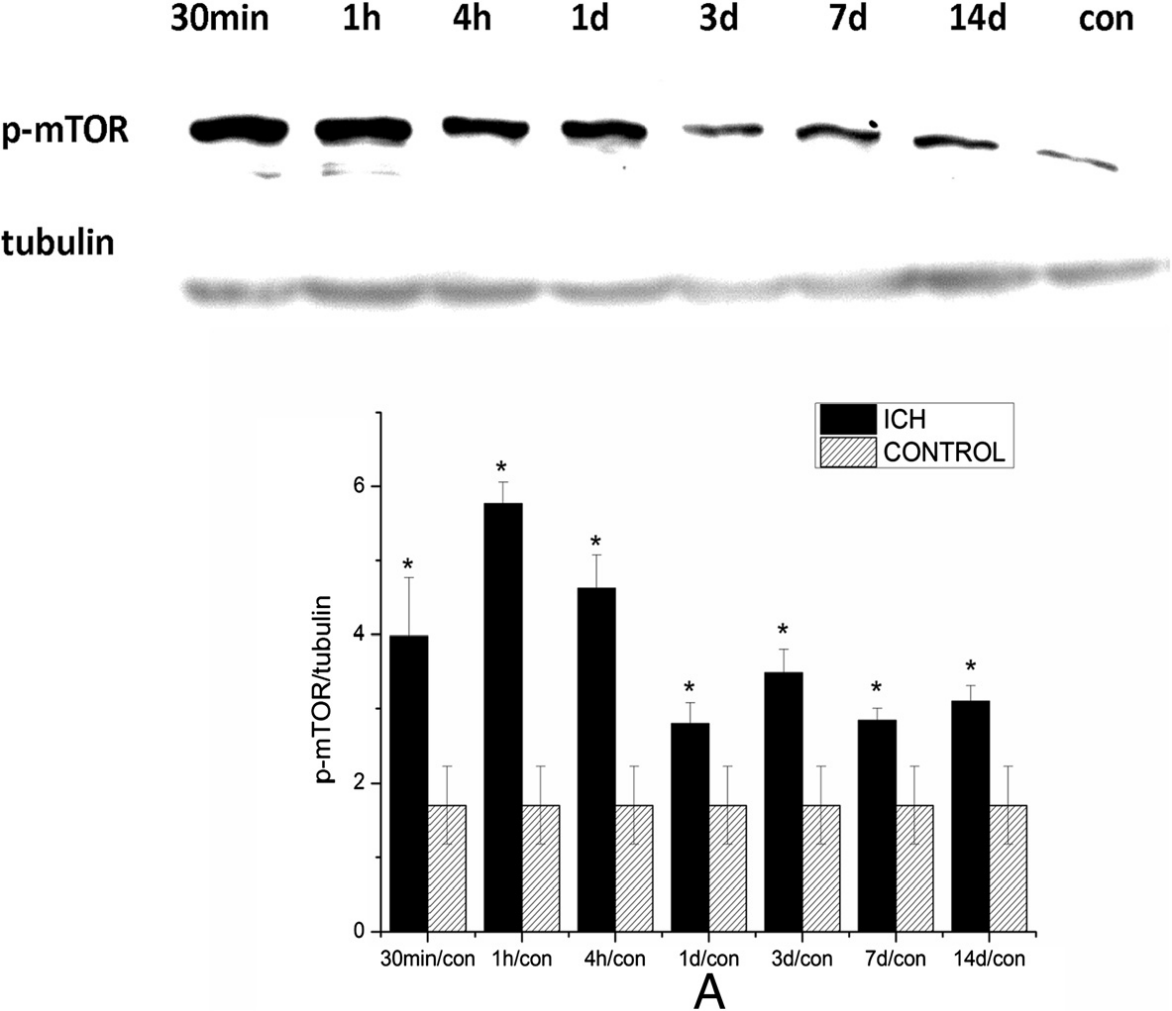
**Mammalian target of rapamycin (mTOR) was activated after intracerebral hemorrhage (ICH). (A)** Protein from the ipsilateral hemisphere was analyzed by western blotting using anti-p-mTOR (Ser 2448)., which was was greatly increased at 30 minutes after ICH and lasted up to 14 days after ICH. The level of p-mTOR was normalized to the level of tubulin. *P<0.05.

**Figure 2:**
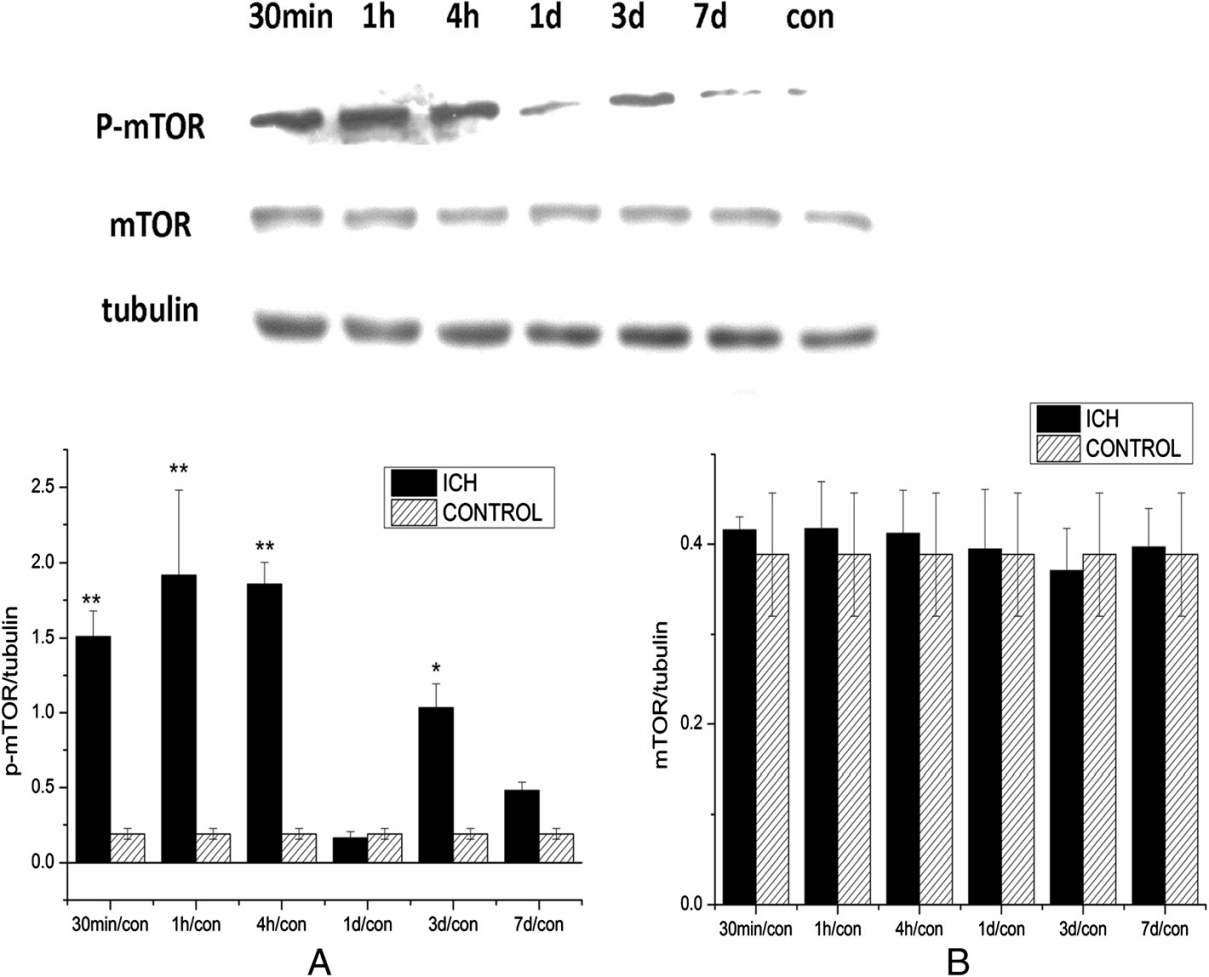
**Increased mammalian target of rapamycin (mTOR) activation in the ipsilateral striatum after intracerebral hemorrhage (ICH). (A)** Protein from the striatum was analyzed by western blotting using anti-p-mTOR (Ser 2448). The level of p-mTOR was normalized to the level of tubulin. The p-mTOR significantly increased at 30 minutes after ICH, and returned to basal level at 1 day after ICH. **(B)** The change in total mTOR was not significant after being normalized to tubulin. *P<0.05; **P<0.001.

**Figure 3:**
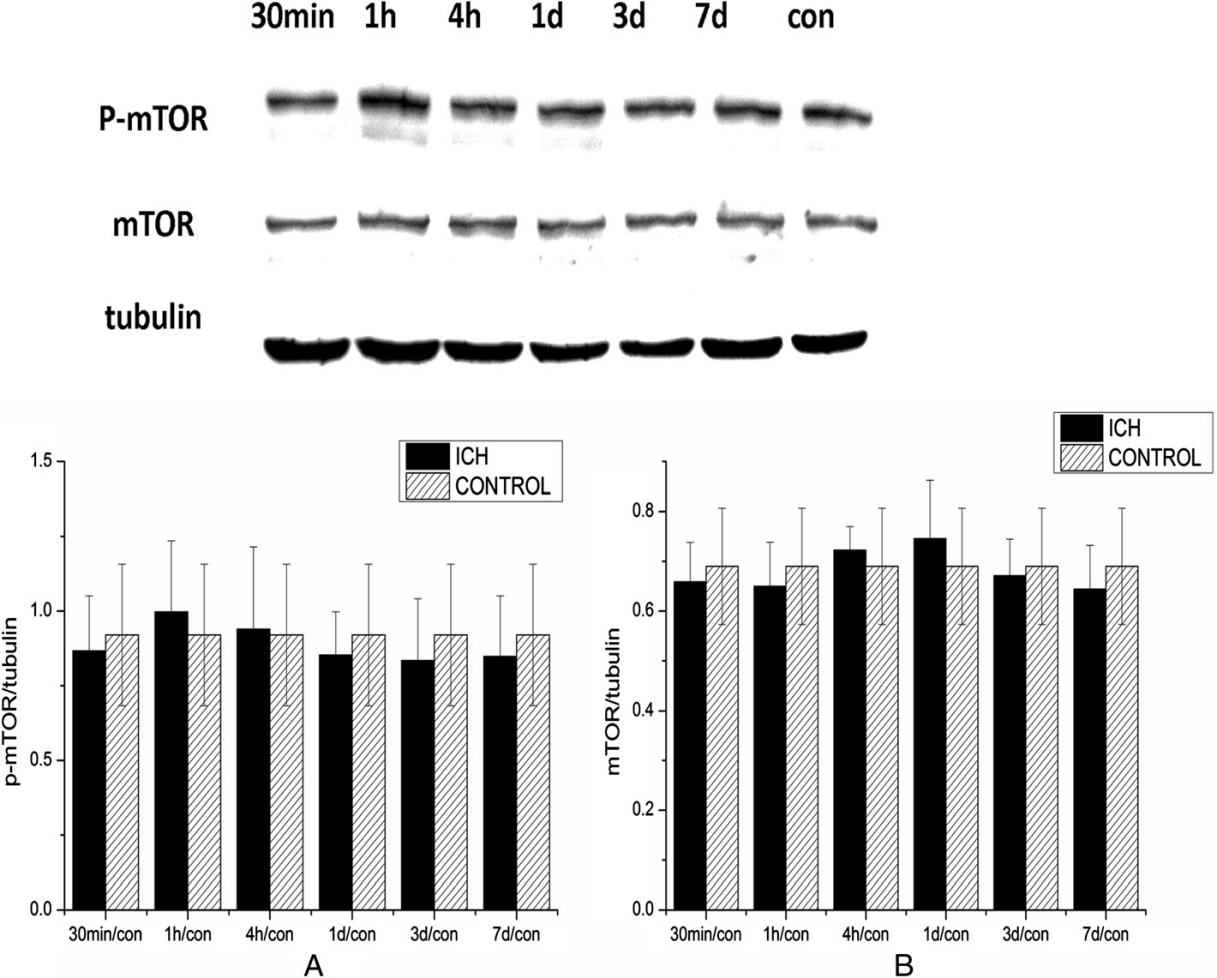
**Mammalian target of rapamycin (mTOR) activation in the ipsilateral cortex after intracerebral hemorrhage (ICH).** The levels of both **(A)** p-mTOR and **(B)** total mTOR did not show significant changes after ICH (after normalization to tubulin).

To study alterations in the mTOR signaling pathway after ICH, protein lysis samples from the ipsilateral hemisphere were separated by electrophoresis and analyzed by western blotting. We found increased phosphorylation of mTOR in the ipsilateral hemisphere of ICH rats from 30 minutes to 14 days after ICH (Figure 1), suggesting that activation of mTOR signaling was both rapid and long-lasting after ICH.To further study the profile of mTOR activation in different regions of the ipsilateral hemisphere, we analyzed the protein from the striatum surrounding the hematoma and cortex (both regions extended 2 mm around the needle tip) by western blotting. We observed that mTOR phosphorylation in the ipsilateral striatum was significantly increased at 30 minutes, peaked at 1 hour, and returned to basal level about 1 day after ICH (Figure 2). The level of total mTOR was not significantly changed after ICH., and the mTOR activation level in the cortex was not significantly activated after ICH (Figure 3).

**Figure 4:**
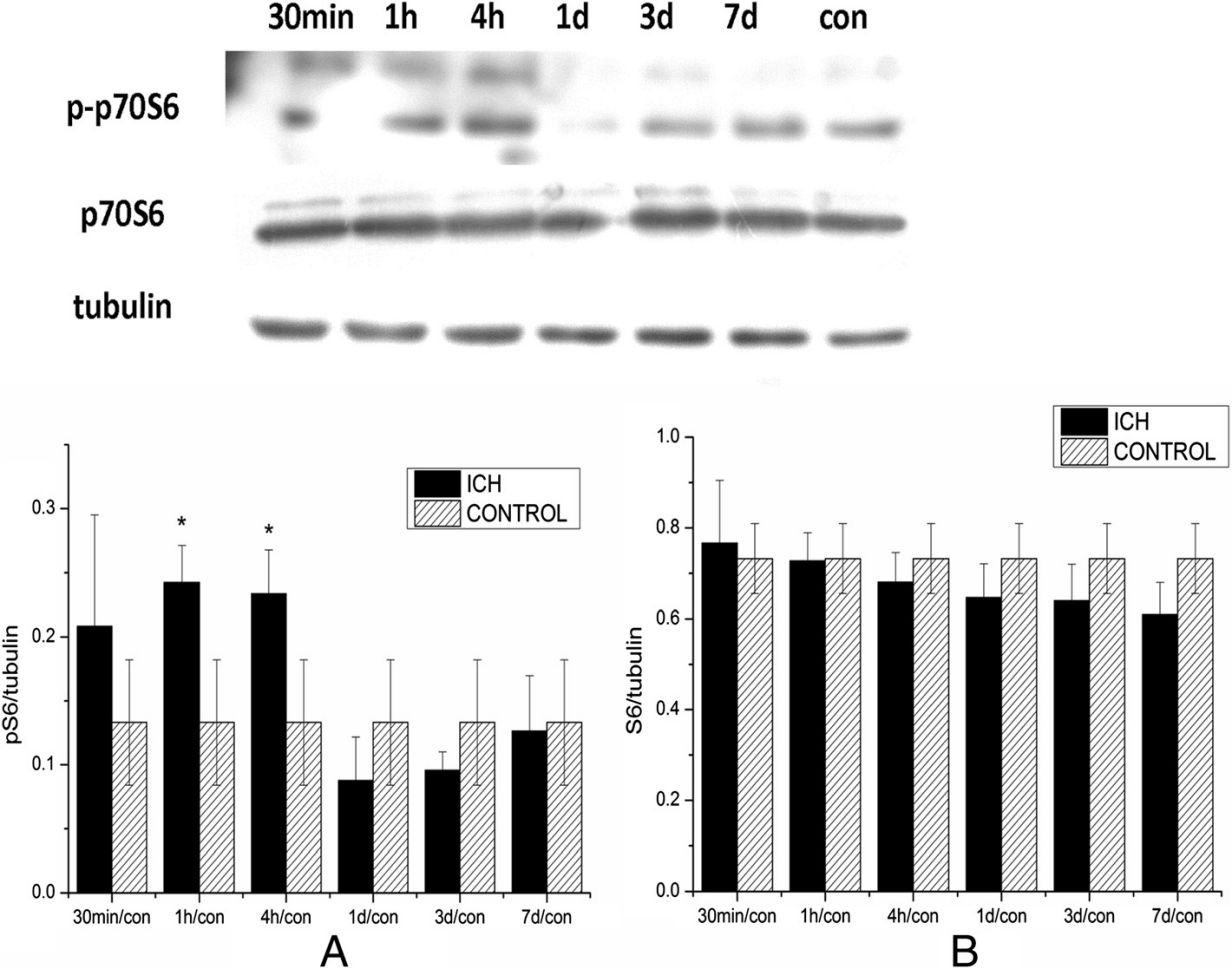
**Increased p70S6 activation in the ipsilateral striatum after intracerebral hemorrhage (ICH). (A)** Protein from the striatum was analyzed by western blotting using anti-p-p70S6 (Thr 389). The level of p-p70S6 was normalized to the level of tubulin. p-mTOR was significantly increased at 1 hour and returned to basal level at 1 day after ICH. **(B)** The change in total p70S6 was not significant after being normalized to tubulin. *P<0.05.

To detect whether mTOR signaling was also activated following ICH, western blotting was performed using anti-p70 ribosomal S6 kinase (p70S6), one of the main downstream targets of the mTOR signaling [29-31]. Compared with the control group, phosphorylation of p70S6 was also activated at 1 hour after ICH (Figure 4), but the level of total p70S6 was not significantly changed after ICH.

### Effect of rapamycin on the neurobehavior of ICH rats

**Figure 5:**
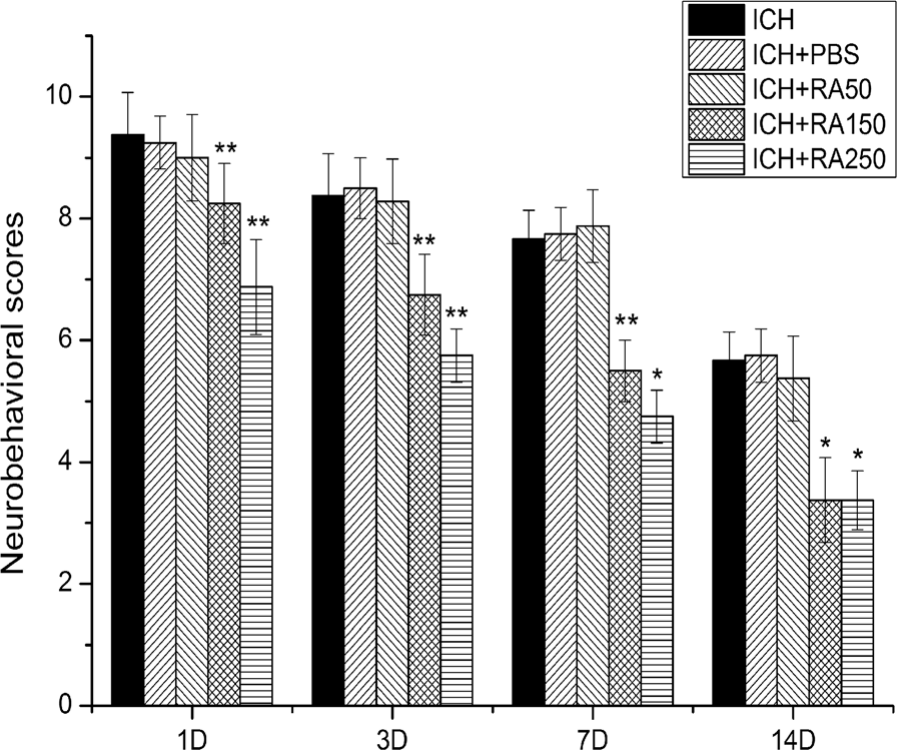
**Rapamycin improves recovery of neurobehavioral function after intracerebral hemorrhage (ICH).** The scores in both the ICH and rapamycin groups were similar before ICH. At 1 hour after ICH, rapamycin-treated groups were injected with rapamycin using different concentrations: 50, 150, 250, and 500 μg/kg. The behaviors were evaluated and compared at 1, 3, 7, and 14 days after ICH. We observed a significant functional recovery in rapamycin-treated groups compared with the ICH group. The mean modified Neurological Severity Score (mNSS) values ± SEM are depicted, *P<0.05; ** *P<*0.001 compared with ICH rats.

To determine the role of mTOR signaling after ICH, rapamycin, an mTOR inhibitor, was injected intraperitoneally at 1 hour after ICH. Neurobehavioral function was evaluated in different groups using the mNSS. Rats were trained 3 days before ICH to assess the normal level (score = 0). At 1 day after ICH, the mean mNSS values were 9.56 ± 0.68 in the ICH group, 9.50 ± 0.50 in the PBS group, 9.00 ± 0.67 in the 50 μg/kg rapamycin-treated group, 8.00 ± 0.67 in the 150 μg/kg rapamycin-treated group, and 7.11 ± 0.74 in the 250 μg/kg rapamycin-treated group (Figure 5). Statistical analysis showed significant differences between the ICH group and the rapamycin-treated groups (except the 50 μg/kg group and 500 μg/kg group). Results for neurobehavioral outcomes at 3, 7 and 14 days after ICH were similar to those given above for 1 day after ICH. There were no significant differences between the ICH group and the PBS group. These results suggest that rapamycin significantly improved the functional recovery of the ICH rats in a dose-dependent manner. However, we found that rats treated with the highest concentration of rapamycin (500 μg/kg) were too sick to be evaluated and six of them died after treatment. Considering that there were no significant differences between the 150 μg/kg and the 250 μg/kg groups at each time point, we propose that the best dose of rapamycin for ICH treatment is 150 μg/kg.

### Rapamycin improves the neurobehavioral deficits in rats after ICH by mTOR signaling

**Figure 6:**
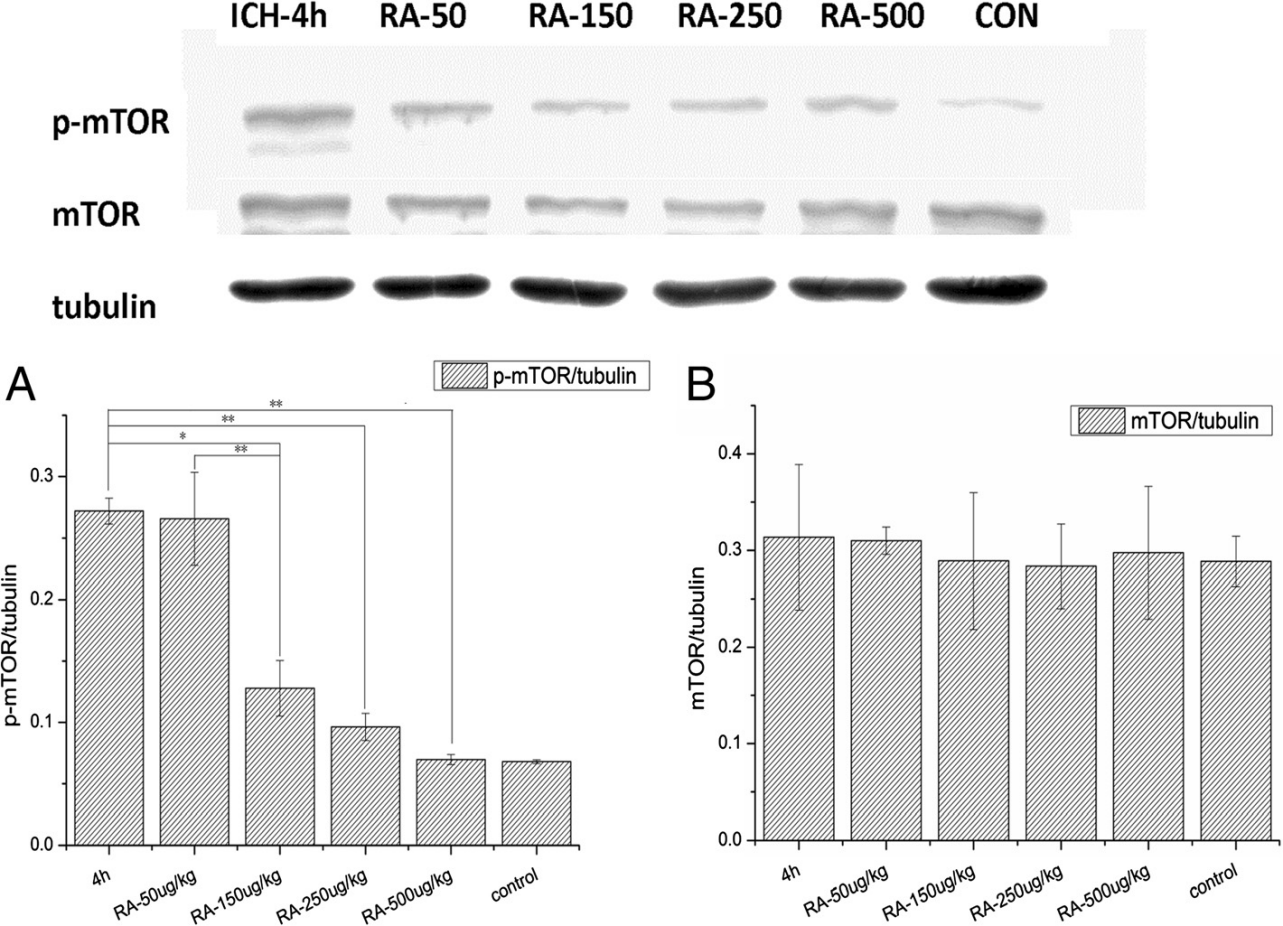
**Rapamycin inhibited p-mammalian target of rapamycin (mTOR) in the striatum. (A)** Compared with the intracerebral hemorrhage (ICH) group (4 h after ICH), significantly lower levels of p-mTOR were observed in the 150, 250, and 500 μg/kg treated groups while there was no significant change in the 50 μg/kg treated group. **(B)** The total mTOR level in rapamycin-treated groups was similar to that of the ICH group. Both p-mTOR and total mTOR were normalized to tubulin. *P<0.05; **P<0.001.

In order to assess whether rapamycin improved functional recovery by inhibiting mTOR signaling, rats were euthanized 4 hours after receiving rapamycin (at various concentrations). As shown in Figure 6, rapamycin treatment (150, 250 and 500 μg/kg, but not 50 μg/kg) significantly reduced the p-mTOR level in the striatum. Although higher concentrations of rapamycin had a stronger inhibition on p-mTOR, there was no significant difference between the groups treated with 150 and 250 μg/kg, further suggesting that 150 μg/kg is the best dose of rapamycin for ICH treatment.

### Rapamycin increases Tregs in the peripheral blood and ipsilateral hemisphere

**Figure 7:**
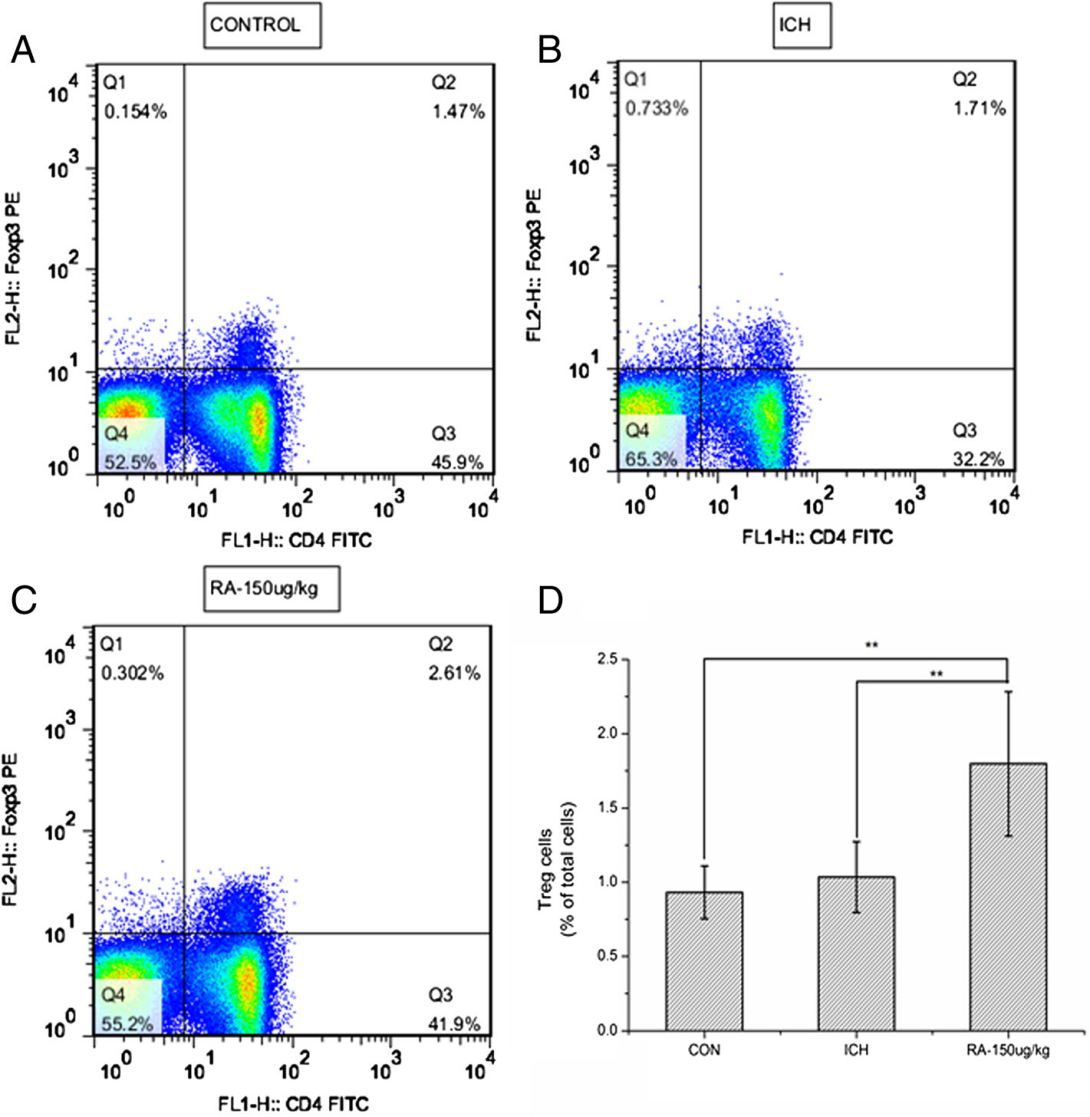
**Rapamycin increased the level of regulatory T cells (Tregs) in the blood.** Dot plots labeled with CD4 and Foxp3 show the blood lymphocytes derived from **(A)** control group, **(B)** intracerebral hemorrhage (ICH) group, and **(C)** 150 μg/kg rapamycin-treated group. **(D)** A statistical graph for the three groups. There were significant differences between the control group and rapamycin-treated groups, and between the ICH and rapamycin-treated groups. *P<0.05.

**Figure 8:**
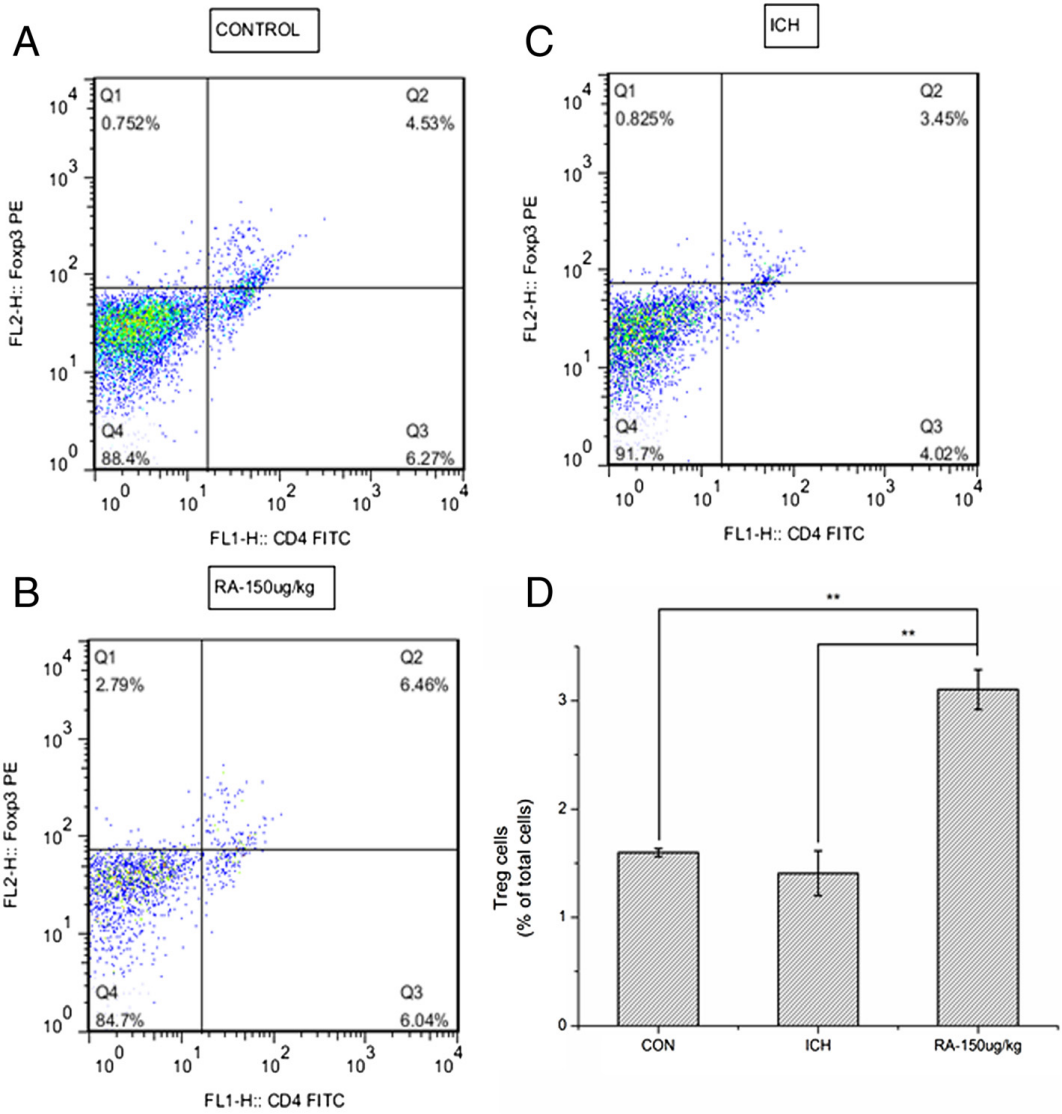
**Rapamycin increased the level of regulatory T cells (Tregs) in the ipsilateral hemisphere.** Dot plots labeled with CD4 and Foxp3 show the brain lymphocytes from **(A)** control group, **(B)** intracerebral hemorrhage (ICH) group, and **(C)** 150 μg/kg rapamycin-treated group. **(D)** A statistical graph for the three groups. There were significantly higher levels of Tregs in the rapamycin-treated group than the control and ICH groups. *P<0.001.

In order to evaluate the effects of rapamycin on the immune response of ICH rats, we separated monocytes from peripheral blood and brain as described previously [28], and analyzed the level of CD4+CD25+Foxp3+ Tregs was analyzed by flow cytometry. The control and ICH groups had similar numbers of CD4+Foxp3+ Tregs in the peripheral blood (0.931 ± 0.145% and 1.035 ± 0.207%, respectively). However, the group treated with 150 μg/kg rapamycin had a significantly higher level of CD4+Foxp3+ Tregs (1.797 ± 0.434%) than the other two groups (Figure 7). Similar to the findings for peripheral blood, CD4+Foxp3+ Tregs in the ipsilateral hemisphere (Figure 8) were increased in the group treated with 150 μg/kg rapamycin (3.103 ± 0.170%), and no significant difference was observed between the control (1.599 ± 0.031%) and ICH (1.407 ± 0.179%) groups. The results indicated that rapamycin could expand CD4+CD25+FoxP3+ Tregs in the peripheral blood and ipsilateral hemisphere.

### Effect of rapamycin on cytokines in peripheral blood and ipsilateral hemisphere

**Figure 9:**
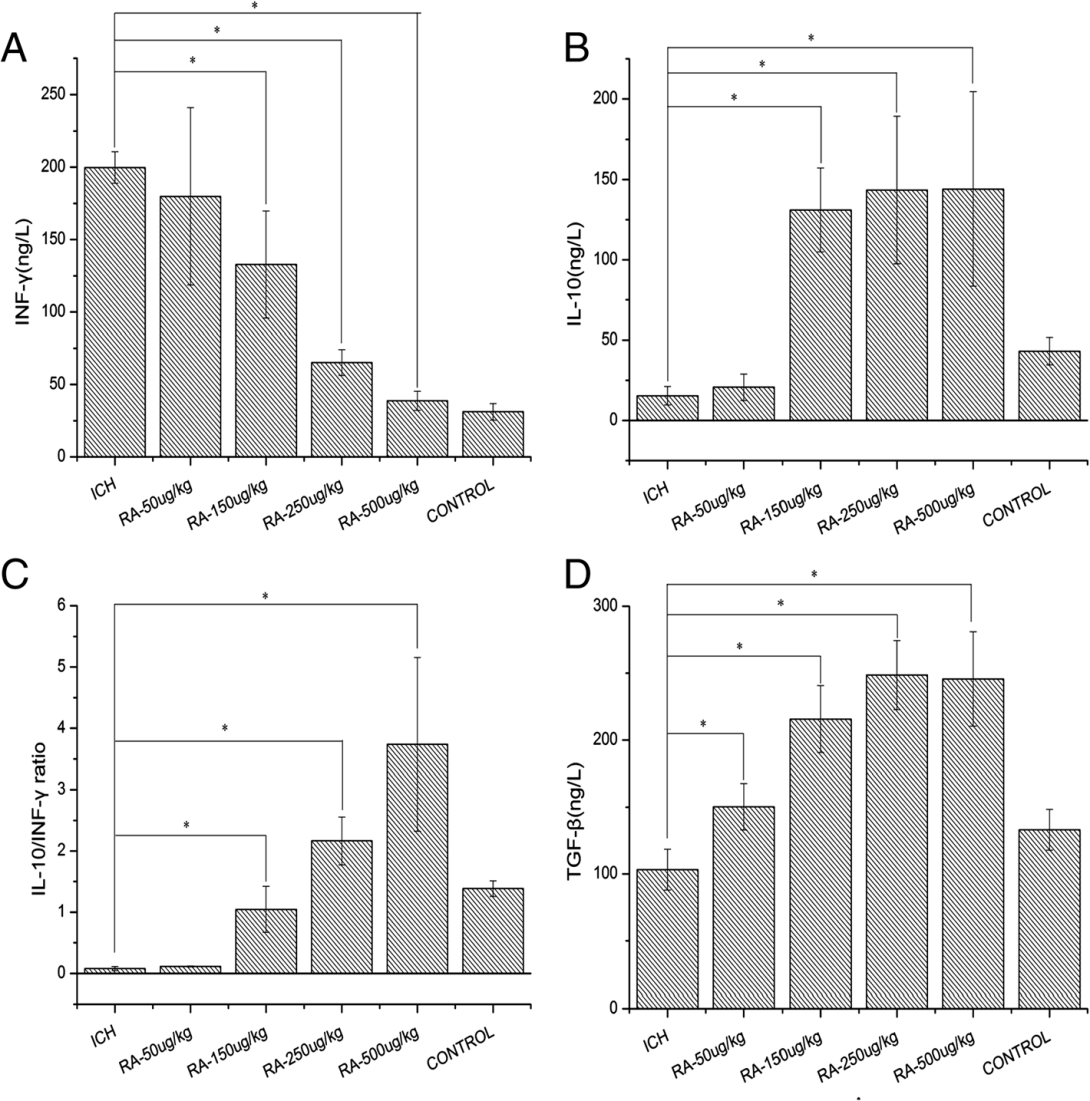
**Levels of cytokines in the serum after rapamycin treatment. (A)** The levels of interferon (IFN)-γ in each rapamycin-treated group were lower than the intracerebral hemorrhage (ICH) group. **(B)** Rapamycin-treated groups presented higher interleukin (IL)-10 expression than the ICH group. **(C)** The ratio of interleukin (IL)-10 to IFN-γ increased after treatment with rapamycin (150, 250, and 500 μg/kg, but not 50 μg/kg). **(D)** Rapamycin increased the level of transforming growth factor (TGF)-β. There were no significant differences between the 150, 250, and 500 μg/kg rapamycin-treated groups. *P<0.05.

**Figure 10:**
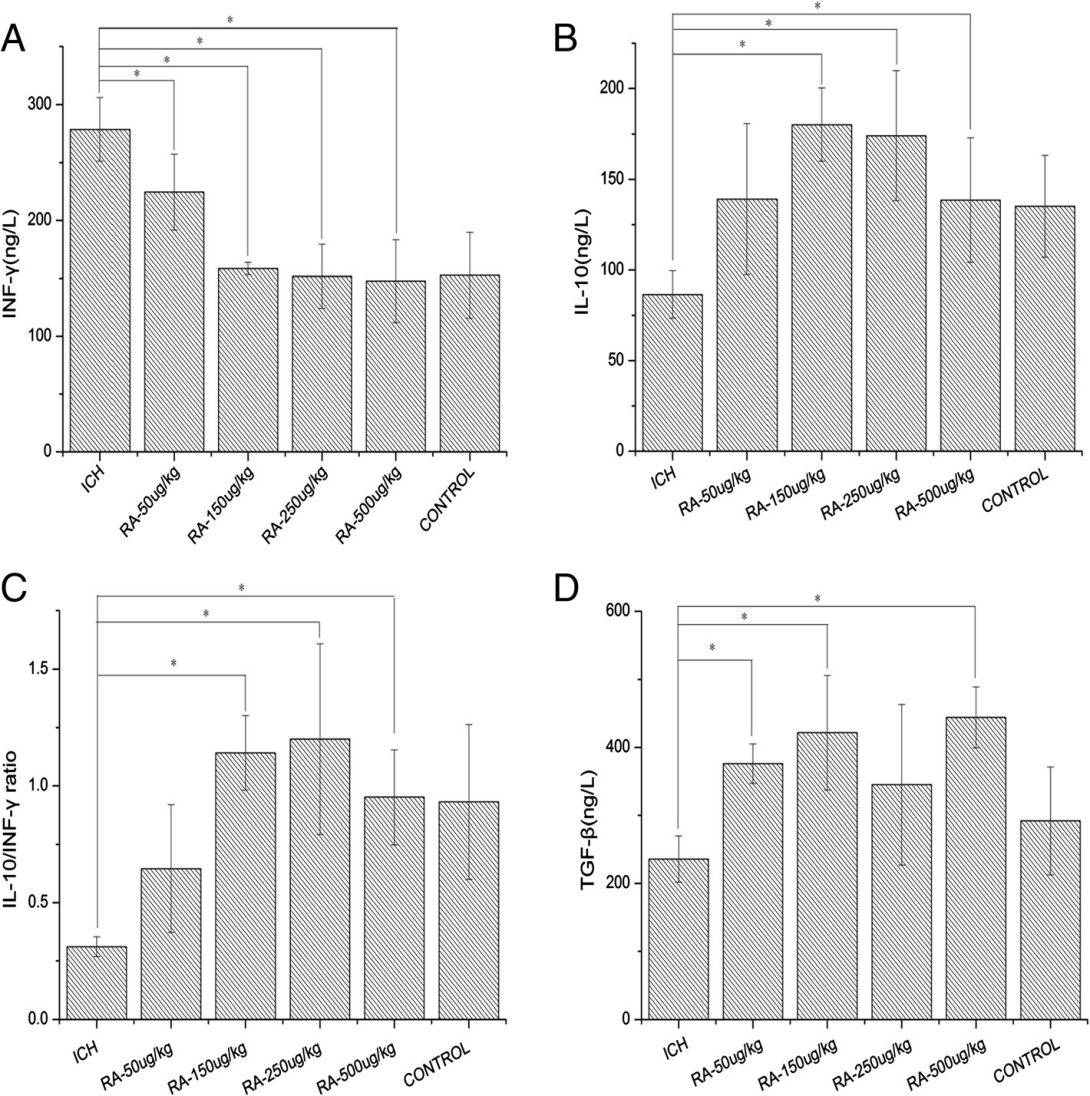
**Levels of cytokines around the hematoma after rapamycin treatment. (A)** Compared with the intracerebral hemorrhage (ICH) group, the levels of **interferon (IFN)-**γ were reduced in the rapamycin-treated groups except for the 50 μg/kg group. **(B)** Rapamycin-treated groups had higher levels of **interleukin (**IL**)**-10 than the ICH group, except for the 50 μg/kg group. **(C)** The ratio of IL-10 to IFN-γ was increased after treatment with rapamycin (150, 250, and 500 μg/kg, but not 50 μg/kg). **(D)** Rapamycin increased the level of transforming growth factor (TGF)-β. *P<0.05.

To further study the effect of rapamycin on cytokines after ICH, we examined the level of IFN-γ and IL-10 in the peripheral blood and around the intracerebral hematoma by ELISA. As shown in Figures 9 and 10, the level of IFN-γ was substantially downregulated both in serum and around the hematoma after treatment with rapamycin (Figures 9A and 10A), whereas the levels of IL-10 and TGF-β were upregulated (Figure 9B,D; Figure 10B,D). The ratio of IL-10 to IFN-γ was significantly higher in rapamycin-treated groups (Figures 9C and 10C). The increased IL-10 and TGF-β, and decreased IFN-γ levels imply that rapamycin modulates inflammatory response in peripheral blood and brain after ICH.

### Effect of rapamycin on immune response in the autologous blood-injection model of ICH

**Figure 11:**
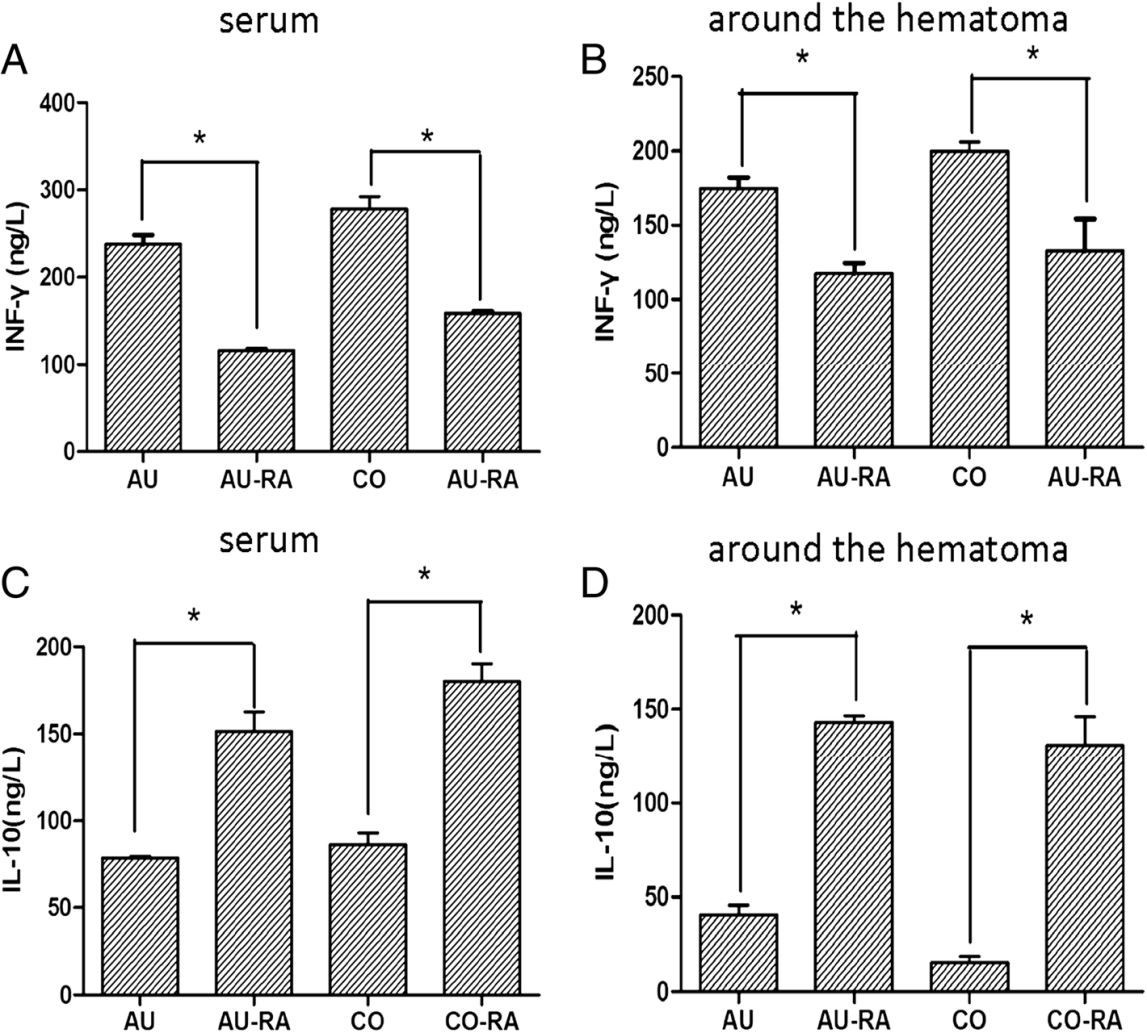
**Effect of rapamycin on the levels of interferon (IFN)-γ and interleukin (IL)-10 at an autologous blood-injection model of intracerebral hemorrhage (ICH). (A, B)** Similar to the collagenase-injection model, the levels of IFN-γ was downregulated after treatment with rapamycin both in serum and around the hematoma in an autologous blood-injection model. **(C, D)** Rapamycin upregulated IL-10 both in serum and around the hematoma. AU, Autologous blood-injection model of ICH, CO, Collagenase-injection model of ICH, RA, Rapamycin. *P<0.05.

Considering that collagenase can induce some cerebral inflammation, we explored the effect of rapamycin (150 μg/kg) on immune response in the autologous blood-injection model of ICH. We found that rapamycin also decreased the level of IFN-γ and increased the level of IL-10 both in serum and around the hematoma in the autologous blood-injection model (Figure 11). Although the level of IFN-γ was higher in the collagenase-injection model than in the autologous blood-injection model after ICH, there was no significant difference between the two models (Figure 11A,B), suggesting that collagenase could induce inflammation, but did not significantly increase the level of IFN-γ. These results further suggest the anti-inflammatory effect of rapamycin after ICH.

## Discussion

In the present study, we found evidence that the mTOR signaling was activated 30 minutes after ICH, and returned to its basal level 1 day after ICH. The increased p-mTOR was predominantly located around the hematoma. Rapamycin treatment significantly improved the neurobehavioral deficit after ICH, and increased the number of Tregs, along with the levels of IL-10 and TGF-β, and reduced the level IFN-γ both in peripheral blood and brain. Our study suggests that mTOR improves ICH outcome and modulates immune response after ICH.

mTOR plays a crucial role in regulating many activities, including protein synthesis, cell growth, and cell death. Accumulating evidence indicates that mTOR is dysregulated in various brain diseases, and inhibition of mTOR by rapamycin provides neuroprotective effects. In a mouse model of tuberous sclerosis complex, Zeng *et al*. found that mTOR signaling is widespread and is stimulated by acute seizure activity, and that rapamycin treatment could reduce mossy fiber sprouting and epilepsy [10]. In a TBI model, Chen *et al*. found that mTOR was significantly activated from 30 minutes to 24 hours and may contribute to the deficits in learning and memory after TBI [14], while Erlich *et al*. demonstrated that rapamycin injection administered 4 hours following TBI reduced microglial activation and increased the number of surviving neurons at the site of injury to improve functional recovery [32]. In a model of neonatal hypoxia-ischemia, Carloni *et al*. showed that rapamycin increased autophagy, reduced necrotic cell death, and decreased brain injury [33]. In a dog model of subarachnoid hemorrhage (SAH), Zhang *et al*. found that mTOR signaling was activated in vascular smooth muscle cells, which contributed to cerebral vasospasm following SAH injury, and that rapamycin could inhibit mTOR signaling and attenuate angiographic vasospasm [34]. Consistently, our study indicated that mTOR was upregulated after ICH, and the outcome was significantly improved through inhibition of mTOR by rapamycin. Our data suggest that mTOR may be a potential therapeutic target for ICH.

mTOR plays a key role in the inflammatory and immune responses [35], which can sense and integrate cues from the microenvironment to control activation and development of immune cells, including T cells and microglia. Microglia, innate immune cells residing in the CNS, contribute to initiation and maintenance of cerebral inflammation, release pro-inflammatory mediators that are potentially cytotoxic, and exert detrimental effects when activated permanently [36]. Cinzia *et al*. showed that mTOR selectively controls microglial activation in response to pro-inflammatory cytokines, and appears to play a crucial role in microglial viability [37]. In our study, mTOR was increased around the hematoma, which may relate to the activation of microglia and release of pro-inflammatory cytokines. Injection of rapamycin at different concentrations (including 150, 250, and 500 μg/kg) significantly decreased the level of IFN-γ and increased the levels of IL-10 and TGF-β both in the hematoma and in serum.

IFN-γ is a key regulator of immune and inflammatory responses. In various neural diseases, IFN-γ is produced by infiltrating T cells, and is associated with onset and process of pathology [38]. IFN-γ was shown to induce the expression of TNF, IL-1β, nitric oxide synthase, and oxygen synthase [39,40], all of which could aggravate the neural disabilities after ICH. By contrast, IL-10, an anti-inflammatory cytokine, was shown to limit inflammation in the brain by reducing the synthesis of pro-inflammatory cytokines, and suppressing expression and activation of the cytokine receptor [41]. In addition, Sharma *et al*. found that IL-10 increased neuronal survival after exposure to oxygen-glucose deprivation by inducing phosphorylation of pro-survival pathways such as AKT and STAT-3 signaling *in vitro*[42]. Thus, by upregulating IL-10 and downregulating IFN-γ 4 hours after ICH, rapamycin plays an anti-inflammatory role in the peripheral blood and brain at an early stage, which could decrease brain damage and contribute to neural recovery.

At the same time, TGF-β is a pleiotropic cytokine with a central role in inflammation. In a murine stroke model, injection of TGF-β1 into the lesion border zone greatly reduced infarct bleeding by recruiting bone marrow-derived monocytes/macrophages, which are important for maintaining integrity of the neurovascular unit following brain ischemia [43]. In humans with stroke, levels of TGF-β1 were found to be increased in the ischemic penumbra, which is essential for angiogenesis after stroke [44]. Abundant studies have shown that TGF-β can induce angiogenesis and neurogenesis following stroke [45-47]. Considering the role of TGF-β in angiogenesis and neurogenesis, rapamycin may promote functional recovery after ICH by increasing the levels of TGF-β in the blood and brain, as shown by our study.

Naive CD4+ T cells, when stimulated by antigen, proliferate and differentiate to T helper type 1 (Th1) cells, Th2 cells and Th17 cells, which favor the generation of Tregs [48,49]. In a model of acute experimental stroke, Liesz *et al*. found that depletion of Tregs profoundly delayed brain damage and deteriorated functional outcome after injection of anti-CD25 antibody. They concluded that Tregs were major cerebroprotective modulators of postischemic inflammatory brain damage through IL-10 signaling [19]. In the present study, we found that Tregs were significantly increased in peripheral blood and ipsilateral hemisphere at 3 days after rapamycin injection. Taken together, these results show that the increased Treg numbers and altered cytokines may be associated with improved outcome after repamycin treatment.

## Conclusion

Our study provides the first evidence that the mTOR signaling pathway is abnormally activated in brain after ICH, especially around the hematoma. Secondly, rapamycin treatment given 1 hour after ICH can promote neurologic functional recovery. Lastly, the underlying mechanisms may be the effect of rapamycin in regulating inflammatory and immune responses, including upregulation of IL-10, TGF-β, and Tregs, and downregulation of IFN-γ. Therefore, mTOR may be a novel therapeutic target for ICH.

## Abbreviations

APC: Allophycocyanin; CNS: Central nervous system; FITC: fluorescein isothiocyanate; HRP: horseradish peroxidase; ICH: Intracerebral hemorrhage; IFN: interferon; IL: Interleukin; mTOR: Mammalian target of rapamycin; PE: Phycoerythrin; PVDF: Polyvinylidene fluoride; SAH: Subarachnoid hemorrhage; TBI: Traumatic brain injury; TGF: Transforming growth factor.

## Competing interests

The authors confirm that there are no conflicts of interests.

## Authors’ contributions

QL designed and performed the study, analyzed data, and wrote the manuscript. LG, WLH, JJY, ZXL, and YLZ performed the study and analyzed data. LJH and LHR helped to analyze data. KLJ helped to design study and write the manuscript, QCZG designed the study and obtained funding for the study. All authors read and approved the final manuscript.
